# Global Calibration of Multi-Cameras Based on Refractive Projection and Ray Tracing

**DOI:** 10.3390/s17112494

**Published:** 2017-10-31

**Authors:** Mingchi Feng, Xiang Jia, Jingshu Wang, Song Feng, Taixiong Zheng

**Affiliations:** 1 School of Advanced Manufacturing Engineering, Chongqing University of Posts and Telecommunications, Chongqing 400065, China; jx_199209@163.com (X.J.); fengsong@cqupt.edu.cn (S.F.); zhengtx@cqupt.edu.cn (T.Z.); 2 College of Mechanical Engineering, Chongqing University of Technology, Chongqing 400054, China; donot@cqut.edu.cn

**Keywords:** camera calibration, multi-camera system, ray tracing, glass checkerboard, bundle adjustment

## Abstract

Multi-camera systems are widely applied in the three dimensional (3D) computer vision, especially when multiple cameras are distributed on both sides of the measured object. The calibration methods of multi-camera systems are critical to the accuracy of vision measurement and the key is to find an appropriate calibration target. In this paper, a high-precision camera calibration method for multi-camera systems based on transparent glass checkerboards and ray tracing is described, and is used to calibrate multiple cameras distributed on both sides of the glass checkerboard. Firstly, the intrinsic parameters of each camera are obtained by Zhang’s calibration method. Then, multiple cameras capture several images from the front and back of the glass checkerboard with different orientations, and all images contain distinct grid corners. As the cameras on one side are not affected by the refraction of glass checkerboard, extrinsic parameters can be directly calculated. However, the cameras on the other side are influenced by the refraction of glass checkerboard, and the direct use of projection model will produce a calibration error. A multi-camera calibration method using refractive projection model and ray tracing is developed to eliminate this error. Furthermore, both synthetic and real data are employed to validate the proposed approach. The experimental results of refractive calibration show that the error of the 3D reconstruction is smaller than 0.2 mm, the relative errors of both rotation and translation are less than 0.014%, and the mean and standard deviation of reprojection error of the four-camera system are 0.00007 and 0.4543 pixels, respectively. The proposed method is flexible, highly accurate, and simple to carry out.

## 1. Introduction

Multi-camera systems (MCSs) have many advantages over single cameras because they can cover wider and more complete fields of view (FOVs), which makes MCSs increasingly prevalent in industrial vision measurements [1,2], visual navigation [3,4], and security monitoring [5], etc. With the advantages of flexibility, cost performance and high precision, industrial vision measurement using MCSs has been widely studied in many applications, such as car body-in-white inspections [6], and deformation and displacement measurements [7,8]. The measurement of dimension, shape, and deformation is a dynamic process, so all cameras should observe parts of the surface from different viewpoints simultaneously (one-shot image acquisition) and dynamically reconstruct the 3D shape of the whole object. This kind of MCS includes multiple cameras sharing an overlapping FOV at different orientations. In special cases, these cameras are distributed in the opposite direction. Accurate calibration of multiple cameras is quite significant [9], since the calibration results determine the mapping relationship between world points and their image projections. Generally speaking, the overall performance of the MCS strongly depends on the accuracy of the camera calibration.

The calibration methods of the MCS are divided into two categories: metric calibration and self-calibration. The proposed method, using knowledge of the scene such as the calibration pattern to calculate stable and accurate calibration results, forms a part of metric rather than self-calibration approaches. Several patterns were proposed for multi-camera metric calibration, which can be grouped into three main categories: 3D calibration targets, planar targets, and one-dimensional targets.

A representative calibration scenario of multi-cameras begins by placing calibration target in the overlapping FOV of the cameras to provide a projection relationship between image and world points [10]. The standard calibration target is a planar pattern, such as a checkerboard. Zhang [11] proposed a flexible new technique to easily calibrate single cameras using a planar pattern which had been used in other types of multi-camera calibration [12,13,14,15]. Dong [12] presented an extrinsic calibration method for a non-overlapping camera network based on close range photogrammetry. This method calibrated the extrinsic parameter of multi-cameras using a vast number of encoded targets pasted on the wall. Baker [13] used textures printed on either side of a board to calibrate dozens of cameras. One side of the board was printed with a set of lines, while the other side of the board was printed with a set of boxes with one missing in the middle. Belden [14] described a refractive calibration procedure applied to calibrate MCSs for fluid experiments. This method contributed to volumetric multi-camera fluid experiments, where it was desirable to avoid the tedious alignment of calibration grids in multiple locations and a premium was placed on accurately locating world points. The authors of [15], developed an MCS to measure the shape variations and the 3D displacement field of a sheet metal part during a single point incremental forming operation. The calibration of the multi-cameras determining camera parameters was described in their paper using a planar calibration target. The planar calibration pattern limits the distribution of multiple cameras, especially when multiple cameras are distributed on both sides of the planar pattern. The uneven printed pattern can also affect the accuracy of camera calibration.

**Figure 1:**
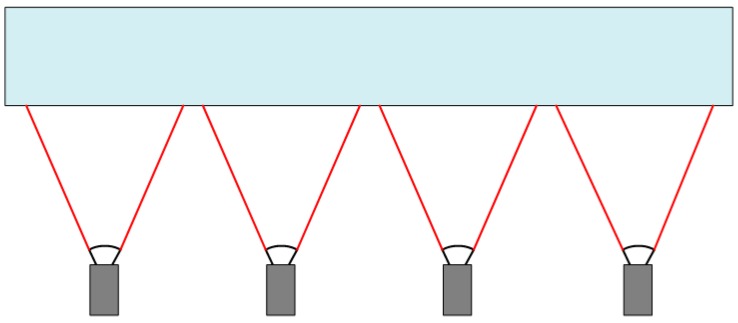
Multi-camera system without an overlapping field of view (FOV).

**Figure 2:**
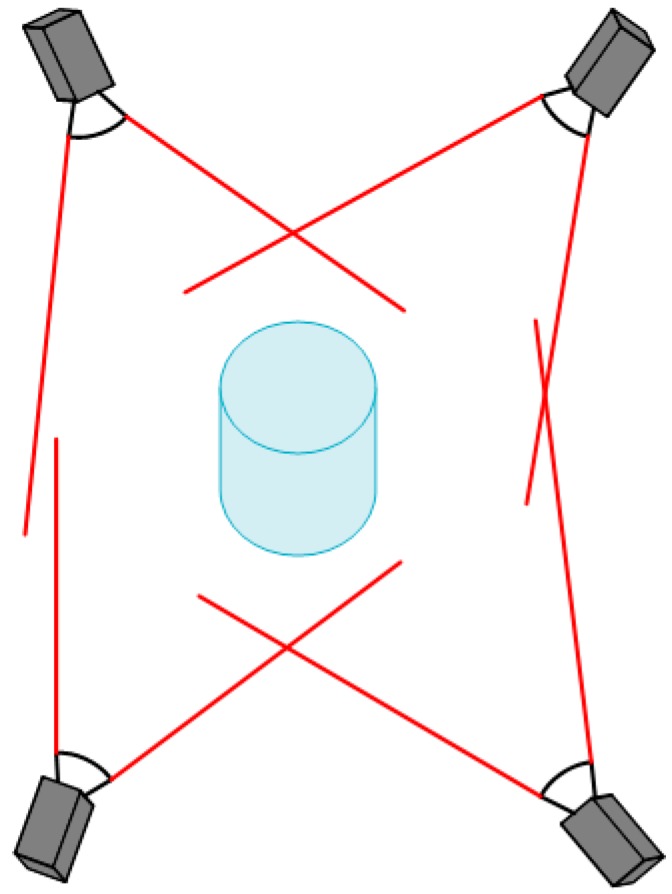
Multi-camera system with an overlapping FOV.

In addition, 1D and 3D calibration targets are also widely used in the calibration of MCSs, such as in Figure 1 and Figure 2. One-dimensional target-based camera calibration was firstly proposed by Zhang [16]. Compared with conventional 2D or 3D target-based camera calibration, the main advantage of 1D target-based camera calibration is that it does not require the 2D or 3D coordinates of markers, which significantly simplifies the manufacturing process of calibration targets. More importantly, without self-occlusion problems, the 1D calibration target can be observed by all cameras in the MCS. The advantage is that all cameras are calibrated simultaneously, which avoids the accumulation of errors when multi-camera calibration is performed in steps or groups. This camera calibration method has been widely used by many MCSs [10,17,18,19,20,21]. However, there are some disadvantages of 1D calibration targets [21]. Firstly, in the construction of the 1D pattern, it is not possible to guarantee the exact linearity of the points, affecting one of the main assumptions of the adopted model. Secondly, another source of error is the tool used to extract the points of the calibration pattern, which cannot achieve the same accuracy of corner extraction in 2D target-based camera calibration.

A typical 3D calibration target is composed of multiple 1D patterns. Shen [10] presented a complete calibration methodology using a novel nonplanar target for rapid calibration of inward-looking visual sensor networks. The calibration target consists of a large central sphere with smaller spheres of different colors mounted on support rods. A flexible method constructing a global calibration target with circular targets was proposed by Gong [2]. Shin [22] described a multi-camera calibration method using a three-axis frame and wand. In this study, the calibration parameters were estimated using the direct linear transform (DLT) method from the three-axis calibration frame. However, the main source of error in this kind of 3D calibration target is attributed to errors of ellipse fitting caused by image noise and lighting conditions. The accuracy of center extraction cannot achieve the same accuracy of corner extraction in a planar pattern [21]. This type of 3D calibration target has the same disadvantages as the 1D calibration target. Another kind of 3D calibration target consists of a multiple planar pattern. Examples are found in the works of Long [23] and Xu [24]. Unfortunately, in MCSs like in Figure 2, it is hard to use this calibration target, which cannot be viewed by all the cameras simultaneously. This 3D calibration target limits the distribution of multiple cameras, which restricts its application.

At the same time, we must realize that the scale of the distributed camera networks grows dramatically while multiple cameras are spread over a wide geographical area. Because the calibration method based on calibration target cannot meet the requirements in many scenarios, the calibration of camera networks purely from the scene has been widely studied. For example, a distributed inference algorithm based on belief propagation had been developed to refine the initial estimate of camera networks [25]. Gemeiner [26] presented a practical method for video surveillance networks to calibrate multiple cameras which have mostly non-overlapping field of views and might be tens of meters apart.

In order to overcome the shortcomings of the foregoing methods, and guarantee high accuracy and convenience of multi-camera calibration, we propose a novel method of global calibration for multiple cameras with overlapping FOVs. This method adopts a planar calibration target made of transparent glass, and the checkerboard pattern is printed on one side of the glass panel. Multiple cameras are distributed on both sides of the calibration target and towards the calibration target. This kind of configuration is useful to get a one-shot 3D shape of the whole object. The cameras in front of the calibration target are not affected by the refraction, and the Zhang’s traditional method can be used to calibrate the intrinsic and extrinsic camera parameters. However, the cameras in the rear of the calibration target are influenced by refraction, and the direct use of Zhang’s method will cause a calibration error. The refraction of glass will affect the accuracy of multi-camera calibration results. This proposed method uses refractive projection model and ray tracing to eliminate the error of refraction. Based on the 3D position accuracy of the corner point on the glass checkerboard being as high as 0.0015 mm, the proposed multi-camera calibration in this paper can achieve high-accuracy and flexibility.

The remainder of this paper is organized as follows: Section 2 introduces the basic mathematical model of the MCS and ray tracing. In Section 3, the proposed calibration method of multi-cameras based on the refractive projection model and ray tracing is described. Section 4 presents a series of experiments (synthetic and real data) to verify the feasibility and accuracy of the proposed approach. A single-camera experiment verifies feasibility of the refractive projection model and calibration of extrinsic camera parameters. A two-camera experiment confirms the accuracy of calibration of extrinsic camera parameters and refractive index. A four-camera experiment verifies the performance of our method used in the actual MCS. The conclusions are indicated in Section 5.

## 2. Mathematical Model of Camera and Ray Tracing

This section briefly introduces the basic concepts used in the calibration of single camera and the MCS. Then, the refractive projection model and ray tracing used in this paper will be described.

### 2.1. Camera Model

An ideal camera is modeled by the pinhole imaging. The relationship between a 3D point in world coordinates and the same point in camera coordinates is approximated by means of the rotation matrix and transformation matrix, as shown in Equation (1).

(1) $$\left[\begin{array}{c} X_{C}\\ Y_{C}\\ Z_{C} \end{array}\right]=R*P+T=R*\left[\begin{array}{c} X\\ Y\\ Z \end{array}\right]+T$$

The projection of the point in camera coordinate on the image is $p={\left[u,v\right]}^{T}$, which obeys Equation (2).

(2) $$\begin{array}{ccc} \lambda \left[\begin{array}{c} u\\ v\\ 1 \end{array}\right]=K\left[\begin{array}{c} X_{C}\\ Y_{C}\\ Z_{C} \end{array}\right] & \mathrm{with} & K=\left[\begin{array}{ccc} f_{u} & \gamma & u_{0}\\ 0 & f_{v} & v_{0}\\ 0 & 0 & 1 \end{array}\right] \end{array}$$

where $P={\left[X,Y,Z\right]}^{T}$ are the world coordinates of a 3D point, ${\left[X_{C},Y_{C},Z_{C}\right]}^{T}$ are the camera coordinates, and ${\left[u,v\right]}^{T}$ are the pixel image coordinates. λ denotes a nonzero scale factor. ${\left[u_{0},v_{0}\right]}^{T}$ denote the principal point in the imaging plane with the unit of pixel. K is the matrix of the intrinsic parameter. $f_{u}$ and $f_{v}$ represent the focal length in pixels along the image axes u and v, respectively, while γ is the skew coefficient defining the angle between the u and v pixel axes. R and T, called the extrinsic parameters, are the rotation matrix and the translation vector from the world coordinate frame to the camera coordinate frame, respectively.

However, the real camera projection is not ideal, particularly when a commercial lens is used. Therefore, the lens distortion on the imaging has to been taken into account. Commonly, only a first-order or second-order distortion model is adopted to correct the radial distortion [11,27,28]. More rigorously, the radial distortion and tangential distortion should be adopted to correct the lens distortion [9,29]. After considering the lens distortion, the new normalized point coordinates ${\left[x_{d},y_{d}\right]}^{T}$ are defined as follows.

The distortion-free and the distorted normalized image coordinates are ${\left[x,y\right]}^{T}$ and ${\left[x_{d},y_{d}\right]}^{T}$, respectively.

(3) $$\left[\begin{array}{c} x\\ y \end{array}\right]=\left[\begin{array}{c} X_{c}/Z_{c}\\ Y_{c}/Z_{c} \end{array}\right]$$

(4) $$\left[\begin{array}{c} x_{d}\\ y_{d} \end{array}\right]=(1+k_{1}r^{2}+k_{2}r^{4}+k_{5}r^{6})x_{n}+dx$$

(5) $$\begin{array}{ccc} dx=\left[\begin{array}{c} 2k_{3}xy+k_{4}(r^{2}+2x^{2})\\ k_{3}(r^{2}+2y^{2})+2k_{4}xy \end{array}\right] & \mathrm{with} & r^{2}=x^{2}+y^{2} \end{array}$$

where $1+k_{1}r^{2}+k_{2}r^{4}+k_{5}r^{6}$ is the radial distortion and dx is the tangential distortion. $k_{1}$, $k_{2}$, $k_{5}$ are the coefficients of radial distortion, and $k_{3}$, $k_{4}$ are the coefficients of tangential distortion. We will use $D=\left[k_{1},k_{2},k_{3},k_{4},k_{5}\right]$ to represent the vector of distortion coefficients in this paper.

Based on the descriptions above, a 3D point P in the world coordinate system (WCS) can be projected to a 2D point p in the image coordinate system using the following projection equation:

(6) p=f(K,R,T,D,P)

### 2.2. Refractive Projection and Ray Tracing

**Figure 3:**
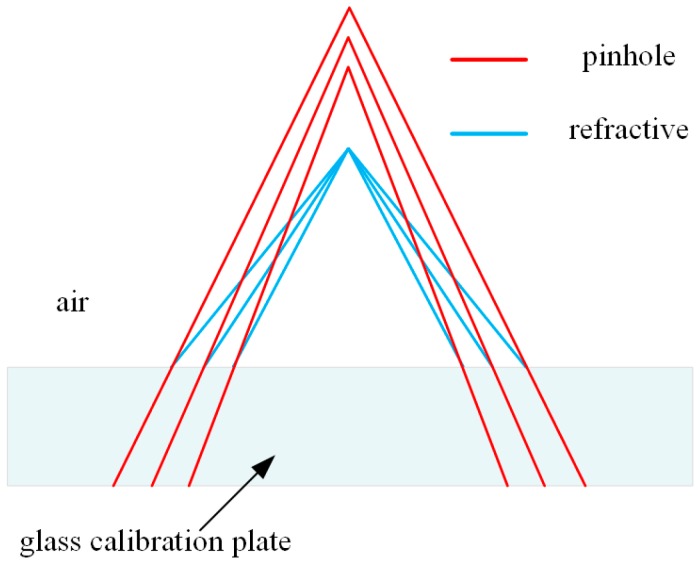
Schematic of imaging through glass using the pinhole and refractive projection model.

Usually, the aforementioned pinhole model can meet the requirements of the camera calibration, but the transparent glass checkerboard is applied in our method. The direct application of the pinhole model between world and image points is erroneous as the refraction of light must be considered in our MCS. As shown in Figure 3, if the rays emanating from the world points are drawn along the path taken in the glass (red line), they do not meet in a single point in the air. In this case, the accurate pinhole model will also lead to error that is exacerbated for cameras when image planes are not parallel to the glass checkerboard. In Belden’s work [14], the image plane is angled relative to the interface, which results in a relatively high calibration error when the pinhole model is applied. The reprojection error using the pinhole model in the experiment of our paper is also the same order of magnitude. The non-ignorable error keeps us from adopting the pinhole model for multi-camera calibration using a glass checkerboard.

In order to eliminate the calibration error caused by refraction, the refraction in the optical paths must be appropriately considered when projecting 3D points into cameras through glass. We need to find the intersection of each ray with the refractive interface between the glass and air, and project the intersection points to the pinhole camera. In this paper, we adopt the ray tracing method proposed by Muslow that initializes the intersection points using an alternating forward ray tracing (AFRT) method [30]. To calculate the intersection of rays with glass surfaces, the points that simultaneously satisfy the equation of a line and the plane equation defining the surface geometry of glass should be solved. A point on a line along the direction of a given ray $\hat{r}$ is defined in Equation (7).

(7) $$X(t)=X_{0}+t\hat{r}$$

The refractive index of air and glass is $n_{1}$ and $n_{2}$, respectively ($n_{2}>n_{1}$). Assume that the refractive index of air is equal to one, and the relative refractive index of glass ($n=\frac{n_{2}}{n_{1}}=n_{2}$) is one of the optimized parameters. The thickness of glass is d. ${\hat{r}}_{i}$ and $\hat{N}$ denote the direction of the incident ray and the normal vector of refractive surface, respectively. The direction of the refracted ray ${\hat{r}}_{t}$ is given by:

(8) $${\hat{r}}_{t}=n{\hat{r}}_{i}+\left[n\hat{N}\cdot {\hat{r}}_{i}-\sqrt{1-n^{2}\left[1-{\left(-\hat{N}\cdot {\hat{r}}_{i}\right)}^{2}\right]}\right]\hat{N}$$

**Figure 4:**
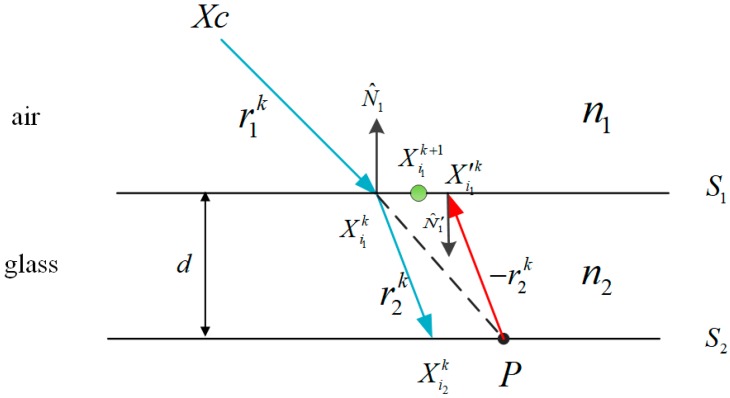
Schematic of ray tracing method.

Figure 4 depicts the algorithm of ray tracing in order to find the intersection of rays with a planar glass surface. The procedure of ray tracing is described as follows:

1. The procedure is initialized by k=1. $r_{1}^{k}$ denotes the direction of the line connecting the camera center $X_{C}$ and the 3D point P. We can find the intersection of $r_{1}^{k}$ and $S_{1}$ at the point $X_{i_{1}}^{k}$.
2. When $n_{1}$ and $n_{2}$ are known, we can find the $r_{2}^{k}$ using the Equation (8), which intersects $S_{2}$ at the point $X_{i_{2}}^{k}$.
3. The ray $-r_{2}^{k}$ is projected from *P* to interface $S_{1}$, and parallel to $r_{2}^{k}$ but opposite in direction.
4. Finally, the ray $-r_{2}$ is intersected with $S_{1}$, resulting the point $X_{i_{1}}^{{}^{\prime }k}$.
5. If the distance $\Delta X_{i_{1}}^{k}=\left|X_{i_{1}}^{k}-X_{i_{1}}^{{}^{\prime }k}\right|$ between the $X_{i_{1}}^{k}$ and $X_{i_{1}}^{{}^{\prime }k}$ is larger than the tolerance, the above procedures will be reiterated, and the point at $\frac{1}{2}\left(X_{i_{1}}^{k}+X_{i_{1}}^{{}^{\prime }k}\right)$ is defined as $X_{i_{1}}^{k+1}$. Otherwise, the optimal solution of the intersection of $r_{1}^{k}$ and $S_{1}$ is found.

In addition to the intrinsic and extrinsic parameters of the camera, the main parameters affecting the projective ray include the refractive index and thickness of refraction glass. The thickness of the glass can be accurately measured. Because the refractive index of different types of glasses is different, the refractive index is considered as unknown. Through the above discussion, Equation (6) can be converted to Equation (9) with refraction.

(9) $$p_{r}=f_{r}(K,D,R,T,P,n)$$

where $p_{r}$ and $f_{r}$ represent the image points generated by the refraction and the refractive projection model, respectively.

## 3. The Proposed Calibration Method

### 3.1. Multi-Camera Calibration Based on Refractive Projection

**Figure 5:**
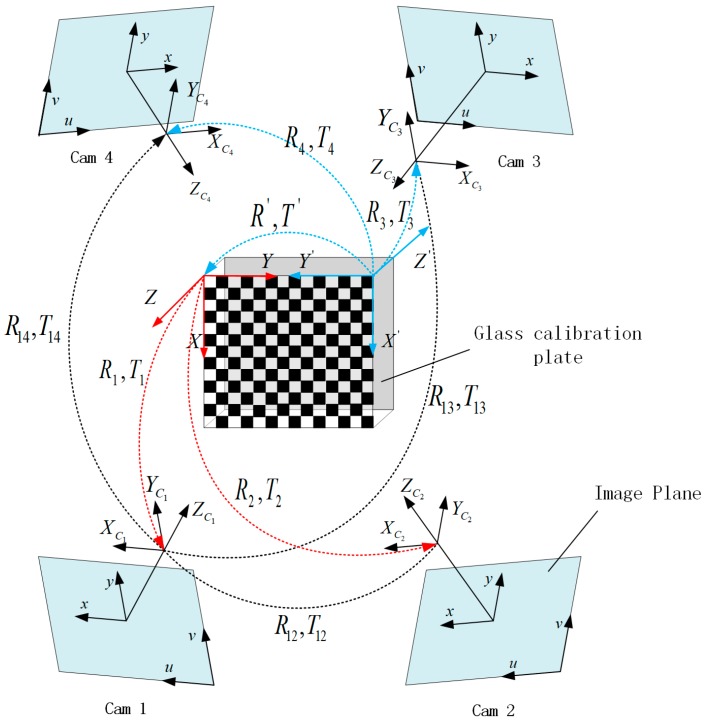
The rotation and translation of the four-camera system.

In the previous section, we introduced the camera model and the refractive projection, which are combined to calibrate the MCS in this section. In our work, the single camera model is extended to the modeling and calibration of a MCS made up of more than two cameras. Without loss of generality, the MCS will be explained by the particular case of a four-camera system, which is also used in the calibration experiments described in the present paper. The MCS is shown in Figure 5, and the object in the center is the glass calibration plate. One side of the glass is printed with a checkerboard pattern, which can be seen from both sides of the calibration plate. Four cameras are distributed on both sides of the calibration plate. These cameras are grouped into two pairs: pair I, including cameras 1 and 2, and pair II, including cameras 3 and 4. The cameras of pair I directly project the 3D point on calibration plate to image without refraction (Equation (6)), while the cameras of pair II are for imaging through the reflection of glass (Equation (9)), which can lead to the calibration errors. The errors can be eliminated by the above refractive projection model and the ray tracing method. Because each camera needs to calculate the initial estimation of extrinsic parameters respectively, the major WCS (red) is fixed on the upper left corner of the pattern of the non-refractive side, and the auxiliary WCS (blue) is fixed on the other side of the pattern with refraction. $R^{\prime }$ and $T^{\prime }$ denote the rotation and translation between the two WCSs.

For an MCS, during the calibration procedure, m (i=1,2,…,m) images of the calibration plate are taken from each camera at different orientations. For each image, n (j=1,2,…,n) object points are recognized by the program. In this system, l (k=1,2,…,l) represents the number of cameras. $K_{k}$ and $D_{k}$ respectively represent the intrinsic camera parameters and distortion coefficients of the kth camera. $R_{ki}$ and $T_{ki}$ denote the rotation matrix and translation vector of the ith position of calibration plate relative to the kth camera. $p^{kij}$ is the projection of the jth 3D point on the ith image of the kth camera without refraction. $p_{r}^{kij}$ denotes the projection of the jth 3D point on the ith image of the kth camera with refraction. The imaging functions are shown as follows.

(10) $$p^{kij}=f(K_{k},D_{k},R_{ki},T_{ki},P_{j})$$

(11) $$p_{r}^{kij}=f_{r}(K_{k},D_{k},R_{ki},T_{ki},P_{j},n)$$

The cameras distributed on both sides of calibration plate use two projection model to solve their extrinsic camera parameters, which are relative to the major WCS or auxiliary WCS. The rotation and translation of each camera need to be aligned to the major WCS. Camera 1 is set as the master camera. The rotation and translation of each camera relative to the master camera is obtained as follows:

(12) $$\{\begin{array}{c} R^{1k}=R_{ki}*R_{1i}\\ T^{1k}=T_{ki}-R^{1k}*T_{1i} \end{array}(\mathrm{without}\;\mathrm{refraction})$$

(13) $$\{\begin{array}{c} R^{1k}=R_{ki}*{\left(R_{1i}*R^{\prime }\right)}^{-1}\\ T^{1k}=T_{ki}-R^{1k}*(R_{1i}*T^{\prime }+T_{1i}) \end{array}(\mathrm{with}\;\mathrm{refraction})$$

$R_{1i}$, $T_{1i}$ are the extrinsic parameters of the master camera and $R^{1k}$, $T^{1k}$ are the relative extrinsic parameters of other cameras relative to the master camera.

### 3.2. Solving Intrinsic Camera Parameters and Initial Estimation of Extrinsic Camera Parameters

The intrinsic parameters of the MCS are obtained by Zhang’s method. Because the positioning accuracy of the 3D point of calibration target is as high as 0.0015 mm, the calibration results are relatively accurate. Before the extrinsic parameters of the system are optimized, it requires an initial estimation of extrinsic camera parameters, which can be obtained using the DLT method described by Hartley [31] or the theory of multi-layer flat refractive geometry presented by Agrawal [32]. The DLT method can only be used when the thickness of glass is relatively small, otherwise the initial estimation of extrinsic parameters will deviate significantly from the truth. The initial estimation of extrinsic parameters gives no consideration to lens distortion and glass refraction, so nonlinear refinement must be applied to the initial estimation aiming at improving accuracy. The best estimate of the camera parameters can be obtained by nonlinear refinement based on the maximum likelihood criterion, such as the Levenberg–Marquardt algorithm. The maximum likelihood estimate for our proposed method can be written as Equation (14).

(14) $$\underset{R_{1i},T_{1i},R^{1k},T^{1k},n}{\min}\sum_{k}^{l}\sum_{i}^{m}\sum_{j}^{n}\left((1-w){\left\|x^{{}_{kij}}-p^{{}_{kij}}\right\|}^{2}+w{\left\|x_{r}^{{}_{kij}}-p_{r}^{{}_{kij}}\right\|}^{2}\right)$$

Equation (14) shows minimization of the sum of the reprojection error, which is a 2D Euclidean distance between the projected points based on Equations (10) and (11) and the actual image points. $x^{kij}$, $p^{kij}$ are the measured image point and the predicted image point without refraction, and $x_{r}^{kij}$, $p_{r}^{kij}$ are the measured image point and the predicted image point with refraction. w is the refraction flag. The value 0 of w indicates the projection without refraction, while 1 means the projection with refraction.

A 3D point and corresponding image point can provide two independent equations. Assuming a l-camera system is applied, each camera takes m image of calibration target, and the calibration object contains j known 3D points. The parameters of the Equation (14) that need to be solved include 6∗m rotation and translation parameters of the master camera, 6∗(l−1) rotation and translation parameters of each camera relative to the master camera, and the refractive index of the glass calibration target. Therefore, 6∗(m+l−1)+1 parameters are solved by 2lmn equations, which leads to an over-determined system. Taking four-camera system as an example, the calibration target contains 182 known 3D points, and each camera captures 20 images. A total of 29,120 equations are solved for 139 variables. Assume that the image points are corrupted by independent and identically distributed noise, and the maximum likelihood solution of these variables is obtained.

The nonlinear optimization algorithms commonly employed in bundle adjustment routines require evaluation of the Jacobian matrix of the projection function, defined in Equations (10) and (11). The individual camera is independent of other cameras and calibration points. Therefore, the Jacobian matrix tends to be a very sparse matrix. The sparse structure can be exploited in the minimization routine to improve computational performance.

The quality of the camera calibration is evaluated by computing the mean and the standard deviation of the individual reprojection errors, which is the residual that exists after minimizing Equation (14). Assuming that the individual reprojection error is d and N is the number of equations, the evaluation parameter can be set as follows.

(15) $$\bar{d}=\frac{1}{N}\sum_{k}^{N}d_{k}$$

(16) $$\sigma_{d}=\sqrt{\frac{1}{N}\sum_{k}^{N}{\left(d_{k}-\bar{d}\right)}^{2}}$$

### 3.3. Summary

The proposed method combines Zhang’s conventional method and the refractive projection model to realize the calibration of the MCS. The global calibration process works as follows:

(1) Multiple cameras are installed and their FOV covers the same area of the calibration target simultaneously. Intrinsic camera parameters and distortion coefficients of each camera are calibrated independently.
(2) In the overlapping FOV of the MCS, multiple cameras acquire the image of the calibration target from different orientations. Images captured by each camera contain the front or back of the calibration target.
(3) Using the DLT method or the theory of multi-layer flat refractive geometry to obtain the extrinsic camera parameters of each camera relative to their WCS, the extrinsic camera parameters of each camera are unified to the master WCS. The rotation and translation of each camera relative to the master camera are obtained as Equations (12) and (13).
(4) The extrinsic camera parameters of the system and the refractive index of the glass are optimized by the bundle adjustment method and the refractive projection model.

## 4. Experiments and Discussion

The accuracy and robustness of the algorithm discussed in this paper are analyzed using both synthetic and real data. Multiple cameras are usually distributed on both sides of the glass checkerboard and towards the calibration target, so both the direct projection model and refractive projection model are adopted in the proposed calibration method. Since the direct projection model has been verified and applied by many scholars, this article will not discuss it. The experiments mainly analyze the refractive projection model, and the two models are simultaneously applied in the calibration of the MCS. In practice, one camera or multiple cameras (for example two cameras) may be deployed on one side of the measured object. In the experiments of synthetic data and real data, we analyze the accuracy of the refractive projection model, which is applied to acquire the refractive index and the extrinsic parameters of single camera and multiple cameras. The extrinsic parameters of each camera are estimated by the DLT method from images of the planar pattern.

### 4.1. Synthetic Data

The intrinsic parameters and extrinsic parameters of the camera are obtained through the 3D points of the calibration target and the corresponding image points. The image points are obtained by the corner detection algorithm in the real data experiment, but the experiment of synthetic data does not need to verify corner detection algorithm. We directly generate the intrinsic and extrinsic parameters of the camera and space points, and obtain the ideal image points using the direct projection model (Equation (10)) and refractive projection model (Equation (11)). The actual image points have the error of corner detection, and the error is simulated by random error of normal distribution. The random error is added to the ideal image point to simulate the real image point.

The simulated camera’s image size is 2592 × 2048 pixels with the principal point at (1296.5, 1024.5) pixels. The focal length along the u and v direction is $f_{u}=2604$ pixels and $f_{v}=2604$ pixels, respectively. All the distortion coefficients are zero. The skew factor is set to zero. The calibration target is a glass checkerboard with 182 corners (14 × 13) uniformly distributed, and the point interval is 12 mm. The glass checkerboard has a thickness of 4 mm and the refractive index of the glass is 1.5. In the generation of the synthetic data, all the images are captured randomly in the constrained range, which includes the distance between camera and object being 300-400mm, and the angles between camera coordinates and world coordinates being $\alpha ={\left(180\pm 15\right)}^{{}^{\circ}}$, $\beta ={\left(90\pm 15\right)}^{{}^{\circ}}$, and $\gamma ={\left(0\pm 15\right)}^{{}^{\circ}}$. The world coordinate frame is set on the checkerboard. The basic parameters of the synthetic experiment are basically consistent with the real experiment.

In order to evaluate the robustness of our method with respect to noise, some simulations have been performed, in which noise is added to the ideal image points ranging from 0 to 0.4 pixels. For each noise level, we perform 100 independent trials and each trial contains 20 images. The estimated camera parameters using simulative image points are compared with the ground truth. In this section, the mean relative error of rotation and translation vector is used to assess the calibration accuracy.

If the rotation vector is v=Rodrigues(R), the relative errors of rotation and translation vector are |∇v|/‖v‖ and |∇T|/‖T‖.

In practice, the thickness of the glass plate is known, while the refractive index is unknown, being equal to approximately 1.5. The smaller change of refractive index has less influence on the image projection, and the refractive effect on the calibration result is relatively small compared to the noise. The extrinsic parameters of the single camera are estimated to be divided into two scenarios: with refractive index estimation and without refractive index estimation.

**Figure 6:**
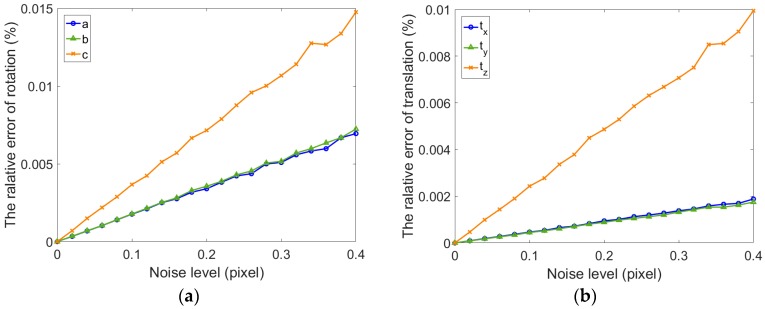
The relative error of extrinsic parameters for one camera without refraction estimation. (**a**) Relative error for the rotation vector; (**b**) Relative error for the translation vector.

**Figure 7:**
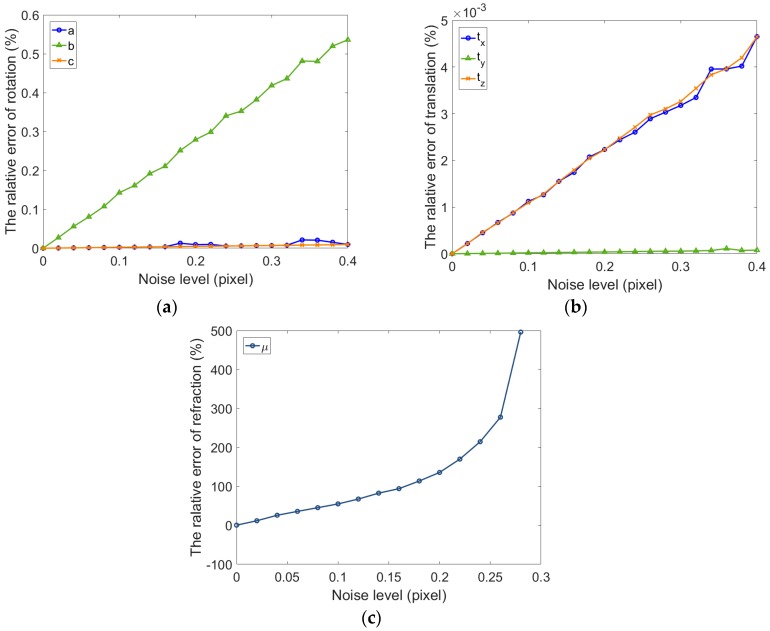
The relative error of extrinsic parameters for one camera with refraction estimation. (**a**) Relative error for the rotation vector; (**b**) Relative error for the translation vector; (**c**) Relative error for the refraction index.

As shown in Figure 6 and Figure 7, whether the refractive index is estimated, the relative errors of rotation and translation vector for single camera gradually increase along with the noise level. The relative error of rotation vector is less than $1.5\times 10^{-6}$ and translation error is less than $1\times 10^{-6}$ when the refraction index is not estimated (μ=1.5). The error of extrinsic parameters using fixed refractive index is more consistent and stable than the error using estimated refractive index. It can be seen from Figure 7a,b that the calibration results in all directions are inconsistent. The growth rate of the error in y direction is inconsistent with the x and z direction. The results are shown in Figure 7c. The error of refractive index increases dramatically, which can be considered incorrect. There is reason to believe that this result is due to an incorrect estimation of the refractive index. When the thickness of glass is small, a single camera cannot accurately estimate the refractive index. The main cause of this problem is that the ray direction is less restrictive. If the cameras can be added in different orientations, the estimated accuracy of refractive index can be improved. Meanwhile, we can also find in Figure 6 and Figure 7 that the extrinsic parameters of the camera are accurate in both cases. When the extrinsic parameters of single camera are estimated, the fixed refractive index can obtain higher accuracy.

In addition to the synthetic experiment of one camera, we have carried out a simulation experiment on multiple cameras using the refractive projection model (taking binocular camera as an example). This experiment is the same as a universal binocular camera because the left camera is a reference camera. The optimized parameters include the rotation and translation of the left camera relative to the world coordinate frame, and the rotation and translation of the right camera relative to the left camera. Meanwhile, the refractive index of glass is estimated and compared with single camera.

**Figure 8:**
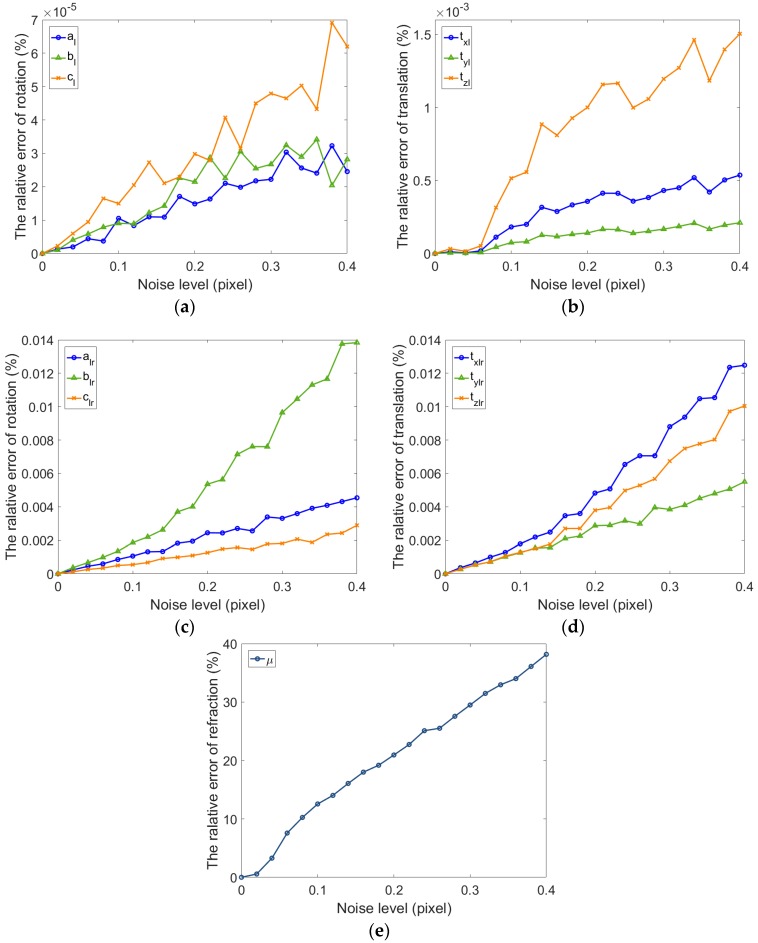
The relative error of extrinsic parameters for binocular cameras with refraction estimation. (**a**) Relative error for the rotation vector of the left camera; (**b**) Relative error for the translation vector of the left camera; (**c**) Relative error for the rotation vector of the left and right camera; (dRelative error for the translation vector of the left and right camera; (**e**) Relative error for the refraction index.

For a binocular camera, Gaussian noise (mean = 0, STD = 0–0.4) is also added to the images of left and right camera, respectively, and then the calibration is conducted with these independent images 100 times. Figure 8 shows the relative error of the extrinsic parameters of binocular camera and the refractive index. It can be seen from the figures that the rotation vector is more accurate than that of the single camera. At the same time, the translation accuracy of binocular camera is lower than for the single camera. Due to the ray constraints of multiple direction of the binocular camera, the precision of estimated refractive index of binocular camera is significantly improved compared with the single camera. Meanwhile, the accuracy of rotation and translation are relatively high.

### 4.2. Real Data

**Figure 9:**
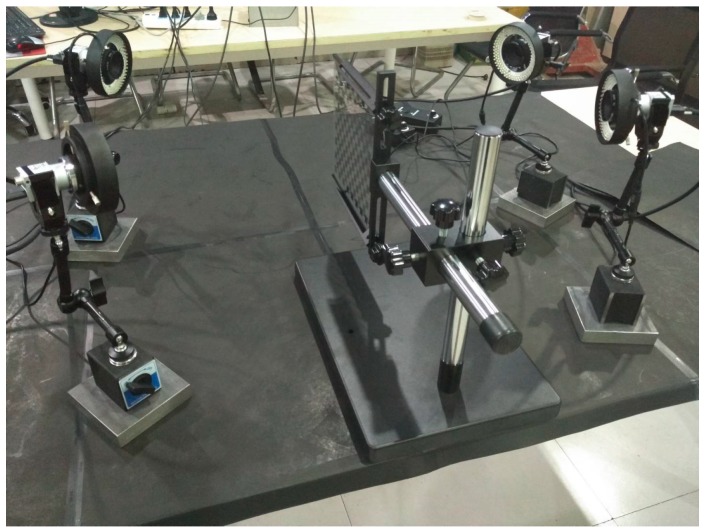
The four-camera system.

**Figure 10:**
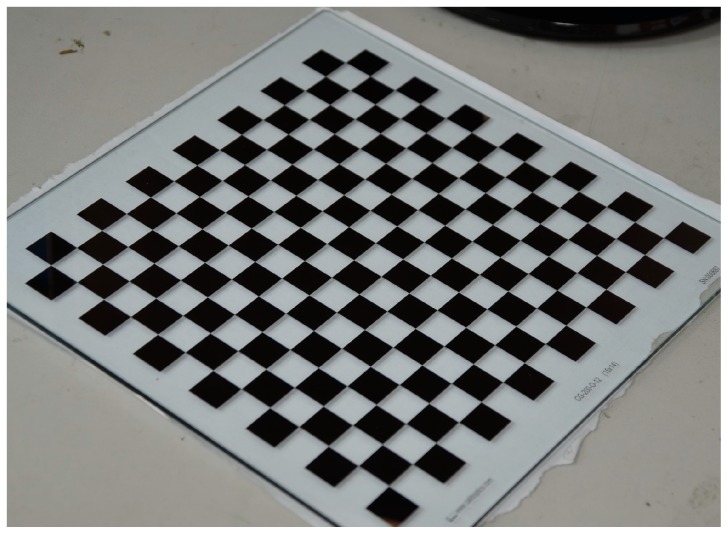
The glass calibration target.

For the experiments with real data, all CMOS cameras (Basler acA2500-60uc) have the same configuration. The focal length of lens is 12.5 mm and the image resolution of the camera is 2590 × 2048 pixels. The four-camera system is presented in Figure 9. As shown in Figure 10, the calibration target is a planar checkerboard with 14 × 13 corner points uniformly distributed. The size of the checkerboard is 200 × 200 mm^2^ and the distance between the adjacent points is 12 mm in the horizontal and the vertical directions. The checkerboard pattern is printed on one side of glass calibration plate with a position accuracy of 0.0015 mm.

It is possible to install one camera or multiple cameras on one side of the measured object. Similar to the synthetic experiment, the experiments of real data will verify the calibration accuracy of one camera, the binocular camera, and the four-camera system. Four cameras are used to perform these experiments using the refractive projection model. Meanwhile, the reflection and overlapping FOV of all cameras can lead to the restriction of positioning the calibration target and the inconvenience of operating the calibration target in the actual application. In order to improve the accuracy and convenience of the proposed method, the intrinsic parameters of all camera are calibrated first and then the extrinsic parameters are calibrated using the proposed calibration method. In the calibration process of intrinsic parameters, the cameras are fixed according to the size of the object and 21 images are taken from different orientations. Table 1 shows the intrinsic parameters of camera 1–4 obtained through Zhang’s flexible calibration method [19]. As Table 1 illustrated, only the distortion coefficients $k_{1}$ and $k_{2}$ are listed.

**Figure 11:**
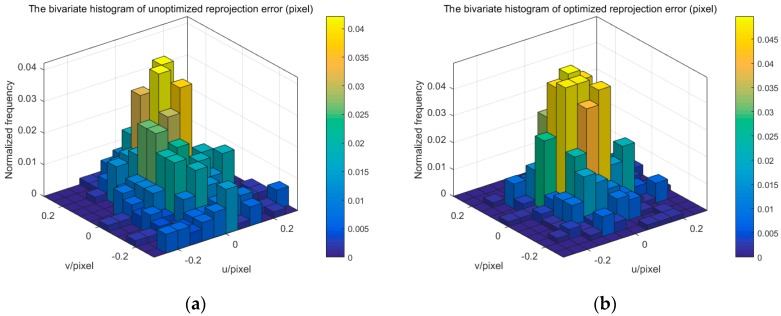
The reprojection error of one camera. (**a**) unoptimized; (**b**) optimized.

**Figure 12:**
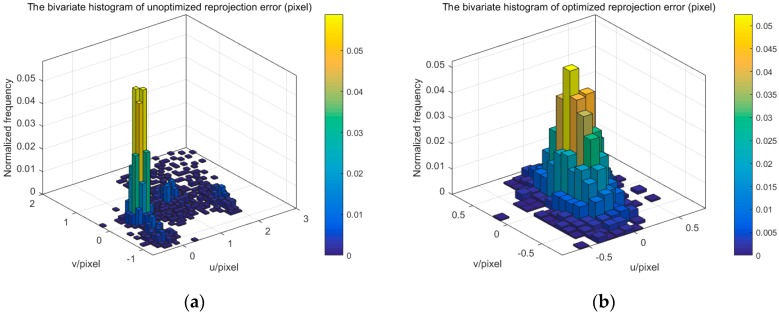
The reprojection error of binocular camera. (**a**) unoptimized; (**b**) optimized.

**Figure 13:**
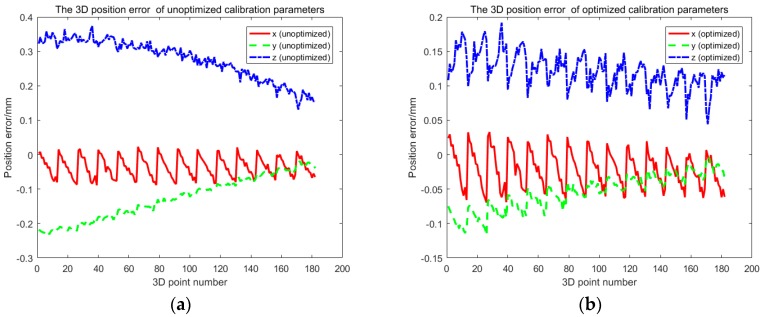
The 3D position error using binocular camera. (**a**) unoptimized; (**b**) optimized.

**Figure 14:**
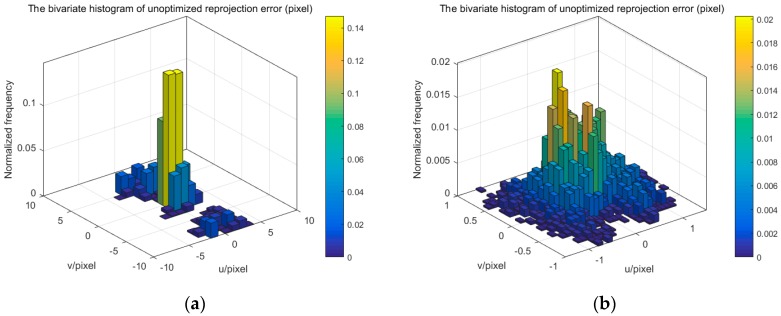
The reprojection error of one camera. (**a**) unoptimized; (**b**) optimized.

The extrinsic parameters of one camera and multiple cameras, and refractive index of glass are solved by using the proposed method. We use the reprojection error of corner point to evaluate the accuracy of camera calibration. Figure 11, Figure 12 and Figure 14 display the bivariate histogram of the unoptimized and optimized reprojection error of one camera, binocular cameras, and four cameras, respectively. The reprojection errors of one camera (camera 4) are shown in Figure 11. It is obvious that the reprojection errors improve significantly through the nonlinear optimization. The initial projection error is larger and the distribution is more dispersed. The optimized error is smaller and the distribution is more concentrated. The mean value and the standard deviation of the initial reprojection errors are 0.0011 pixel and 0.1452 pixel, respectively. After the optimization, the mean value of the reprojection errors is −0.00003 pixels and the standard deviation is 0.0949 pixels. The calibration results of the binocular cameras (cameras 3, 4) are shown in Figure 12. The comparison between the results of refractive calibration and the initial value shows that the bundle adjustment with refractive projection model is more reliable and more accurate. The mean value and standard deviation of reprojection errors change from 0.2842 and 0.6791 pixels to −0.0005 and 0.2213 pixels. The optimized extrinsic parameters of binocular camera are used to calculate the 3D position of the corner point. Then, the position error is calculated based on the 3D position and the theoretical value. As shown in Figure 13, the position error of optimized extrinsic parameters has been reduced to half of the unoptimized one. As can be found from Figure 13, the curve of position error presents a symmetric waveform. We believe that the main reason is that the distortion cannot be completely eliminated, and the imaging will be affected by the distortion coefficient. Therefore, the position error of the center of image is small, while the error of the edge is large. The horizontal coordinate of the figure is the number of points, and the counting mode is increased from top to bottom and from left to right, so it is distributed in a systematic waveform.

In the one-camera and binocular camera system, we only use the refractive projection model. Four cameras are distributed on both sides of the glass calibration plate, which simultaneously uses the direct projection model and the refractive projection model. The four-camera system is used to verify the practicability of our presented method. Figure 14 shows the reprojection error of the four-camera system. The mean value and standard deviation of reprojection error change from −0.3378 and 2.9542 pixels to 0.00007 and 0.4543 pixels, respectively. When the number of cameras is greater than two, the initial extrinsic parameters of the camera are inaccurate. From Figure 12 and Figure 14, it can be seen that the reprojection error of different cameras is not concentrated, resulting in multiple peaks. The binocular camera’s reprojection error ranges from −1 to 3 pixels, while the error of the four cameras ranges from −10 to 10 pixels. After optimization, accurate camera parameters are obtained, so that the reprojection error is reduced, while the multiple peaks of the reprojection error are eliminated, and the distribution conforms to the normal distribution. All this means that the error of extrinsic parameters is reduced. We can also discover that the standard deviation of reprojection errors is basically linear to the number of cameras. The optimized calibration results indicate the stability and accuracy of our proposed method in real data experiments. The relative extrinsic parameters of the four-camera system are reported in Table 2.

### 4.3. Discussion

The above experiments based on synthetic and real data verify the accuracy and effectiveness of the proposed method. This method is applicable to the multi-camera measurement system which can perform a one-shot measurement of the dynamic shape of the whole part. The typical MCS is shown in Figure 5, and the cameras are distributed both sides of the glass calibration plate. Several patterns were designed for multi-camera calibration, which can be grouped into three categories: 1D patterns, 3D target consisting of 1D patterns, and planar patterns. Compared with planar patterns, the disadvantage of other two calibration targets is that it is difficult to guarantee the exact linearity and the extraction accuracy of the points. However, the opaque planar pattern means it is difficult to complete multi-camera calibration and it is easy to generate cumulative errors. With the help of a precision manufacturing technique, a transparent glass calibration target can overcome the above limitations and complete the calibration of the MCS. The position accuracy of corner point on commercial glass calibration plate can reach 0.0015 mm, so it can satisfy the precision requirement of multi-camera calibration. The extrinsic parameters can be optimized in the global coordinates, and the refractive projection model is used to eliminate the refractive effect.

However, the proposed method also shows some limitations. Due to the reflection of glass, the camera’s distribution and the calibration accuracy of multiple cameras are affected. In the calibration process of this paper, a few reprojection errors can occur with abnormal values, which are caused by the reflection. Fortunately, the number of these outliers is very small and they have little impact on the calibration results. Alternatively, we can delete these outliers and reduce the impact on the calibration results. The method can also improve the reflection from the production process. Even if the method is affected by reflection, compared with the existing methods based on 2D or 3D calibration targets [10,33], the mean and standard deviation of the reprojection error based on our method are relatively small. In addition, the calibration method cannot be applied to the multi-camera calibration without an overlapping FOV.

## 5. Conclusions

A typical MCS is installed on both sides of the measured object, which makes it difficult to calibrate the system using the existing camera calibration methods. In this paper, a novel multi-camera calibration method based on glass calibration plates and ray tracing is proposed. Based on the traditional direct projection model, the refractive projection model is developed and the model is applied for multi-camera calibration. Firstly, the mathematical models of refractive projection and bundle adjustment are established with introduction of ray tracing. Then, the intrinsic parameters of each camera are obtained by Zhang’s calibration method and direct linear transformation is used to obtain the initial extrinsic parameters. Finally, the modified bundle adjustment method is applied to optimize the extrinsic parameters of the MCS and the refractive index of glass calibration target. The experimental results of refractive calibration show that the error of the 3D reconstruction is smaller than 0.2 mm, the relative errors of both rotation and translation are less than 0.014%, and the mean and standard deviation of the reprojection error of the four-camera system are 0.00007 and 0.4543 pixels. The experiments performed on synthetic and real data indicate that our proposed method has high accuracy and feasibility.

## Figures and Tables

**Table 1:** The intrinsic parameters of four cameras.

	Camera 1	Camera 2	Camera 3	Camera 4	Uncertainty (3σ)
Focal length	$\left[\begin{array}{c} 2618.29\\ 2618.20 \end{array}\right]$	$\left[\begin{array}{c} 2625.76\\ 2625.61 \end{array}\right]$	$\left[\begin{array}{c} 2617.17\\ 2616.88 \end{array}\right]$	$\left[\begin{array}{c} 2620.34\\ 2620.35 \end{array}\right]$	$\left[\begin{array}{c} 0.49\\ 0.44 \end{array}\right]$
Principal point	$\left[\begin{array}{c} 1290.91\\ 1014.72 \end{array}\right]$	$\left[\begin{array}{c} 1286.45\\ 1001.44 \end{array}\right]$	$\left[\begin{array}{c} 1255.36\\ 1026.86 \end{array}\right]$	$\left[\begin{array}{c} 1293.70\\ 1006.56 \end{array}\right]$	$\left[\begin{array}{c} 0.80\\ 0.73 \end{array}\right]$
Distortion ($k_{1}\;k_{2}$)	$\left[\begin{array}{c} -0.1338\\ 0.1326 \end{array}\right]$	$\left[\begin{array}{c} -0.1356\\ 0.1462 \end{array}\right]$	$\left[\begin{array}{c} -0.1332\\ 0.1360 \end{array}\right]$	$\left[\begin{array}{c} -0.1324\\ 0.1344 \end{array}\right]$	$\left[\begin{array}{c} 0.0008\\ 0.0036 \end{array}\right]$

**Table 2:** The relative extrinsic parameters of the four-camera system.

	Camera 2-1	Camera 3-1	Camera 4-1	Uncertainty (3σ)
Rotation Vector	$\left[\begin{array}{c} 0.0892\\ 0.7389\\ 0.0365 \end{array}\right]$	$\left[\begin{array}{c} 0.1479\\ 3.0076\\ 0.2330 \end{array}\right]$	$\left[\begin{array}{c} 0.0212\\ -2.3761\\ -0.1040 \end{array}\right]$	$\left[\begin{array}{c} 0.0018\\ 0.0026\\ 0.0009 \end{array}\right]$
Translation vector	$\left[\begin{array}{c} -248.9713\\ 1.0768\\ 93.0314 \end{array}\right]$	$\left[\begin{array}{c} -4.3414\\ -81.7511\\ 766.3633 \end{array}\right]$	$\left[\begin{array}{c} 274.9126\\ -42.0589\\ 641.0544 \end{array}\right]$	$\left[\begin{array}{c} 0.1858\\ 0.1280\\ 0.3189 \end{array}\right]$
